# The Effects of Solid Particle Containing Inks on the Printing Quality of Porous Pharmaceutical Structures Fabricated by 3D Semi-Solid Extrusion Printing

**DOI:** 10.1007/s11095-022-03299-7

**Published:** 2022-06-03

**Authors:** Xin-Yi Teoh, Bin Zhang, Peter Belton, Siok-Yee Chan, Sheng Qi

**Affiliations:** 1 School of Pharmacy, University of East Anglia, Norwich, UK; 2 School of Pharmaceutical Sciences, Universiti Sains Malaysia, Penang, Malaysia; 3 School of Chemistry, University of East Anglia, Norwich, UK

**Keywords:** 3D printing, dimensional fidelity, ink rheology, particle size, semi-solid extrusion

## Abstract

**Purpose:**

Semi-solid extrusion (SSE) 3D printing has potential pharmaceutical applications for producing personalised medicine. However, the effects of ink properties and drug incorporation on the quality of printed medication have not been thoroughly studied, particularly for porous geometries. This study aimed to investigate the effects of the presence of solid drug particles in SSE inks on the printing quality of porous structures.

**Method:**

The rheological behaviour of model inks of paracetamol (PCM)-hypromellose (HPMC) with different drug loadings were investigated and correlated to their printing qualities.

**Results:**

For the inks with PCM loading above the drug solubility in which suspended solid drug particulates were present, the results confirmed that PCM loading and particle size significantly affected the ink viscosities at a low shear rate. At a low shear rate, the highest viscosity was identified when the highest drug loading and the smallest PCM particles were incorporated into the inks. However, the results indicated that the SSE printing parameters and printing quality of porous structures (with less porous structural deformation) have no clear correlation with the shear viscosity data, but a strong correlation with the dynamic oscillatory rheology of the inks.

**Conclusion:**

The key rheological parameters including storage modulus, loss modulus and complex viscosity of the ink increased with increasing drug loading for the inks containing solid drug particles. However, decreasing the particle size did not have a clear effect on the oscillatory rheology of the inks which can be potentially used for optimising the SSE 3D printing quality of porous geometries.

**Supplementary Information:**

The online version contains supplementary material available at 10.1007/s11095-022-03299-7.

## INTRODUCTION

Semi-solid extrusion (SSE) 3D printing technology is an additive manufacturing technique where the gel or paste-like materials are deposited layer-by-layer to construct three-dimensional structures. In recent years, SSE 3D printing has been reported for use in potential pharmaceutical applications, such as tablets (1–5), polypills (6–8), chewable dosage forms (9–11) and orodispersible films (12, 13). This technology provides the flexibility for adjusting the pharmaceutical dose that is tailored to individual patients’ needs (14). Research efforts have been made to maximise the drug loading in order to reduce pill size and modify drug release patterns (1, 3, 15, 16). For instance, Khaled *et al*. used SSE 3D printed guaifenesin bilayer tablets with a minimum active pharmaceutical ingredient (API) content of 81% w/w (15), and immediate-release paracetamol tablets with 80% w/w drug loading (3). Cui *et al*. reported SSE 3D printed levetiracetam immediate-release tablets with up to 96% w/w (16) drug loading for patient-tailored medicine. In SSE printing, as the inks contain liquid media, which is removed during post-print drying, the properties of the ink can have a significant impact on the quality of the prints. In addition, in such high dose formulations, drug loading exceeds the drug solubility in the ink, thus and contain solid drug particles.

Solid particles in a viscous liquid are known to affect the rheological and subsequently the flow properties of the liquid (17). In the field of slurry-based ceramic 3D printing by extrusion, particle size distribution and packing in the paste were identified to affect the printing result (18). A similar situation is likely to occur in pharmaceutical printing when using ink containing solid particulates. However, the effects of solid particulates (being either API or excipient) on the SSE 3D printing inks for pharmaceutical applications has not been systemically investigated in the literature. Sieving and particle size control was reported to resolve printing nozzle blockage (1, 19) for pharmaceutical inks, but the effects of particles on the optimisation of the SSE printing and the printing quality of the geometries were not studied.

The aim of this study is to provide new understandings of both the effects of drug loading and particle size of undissolved drug particles on the printing process and the printing quality of the targeted porous structures. To achieve this, a simple binary drug-polymer combination was used as the model and paracetamol (PCM) was selected as the model drug as its aqueous solubility allows investigation of a wide range of the drug loading at below and above saturation concentration of the drug in the ink. Hydroxypropyl methylcellulose (HPMC), also known as Hypromellose, was chosen as the polymer matrix for the inks as it is a standard oral pharmaceutical excipient and frequently utilised as the base in formulating SSE inks (2, 4, 5, 9, 15, 20). The correlations between the rheological properties of the inks and the printing qualities of porous structures were studied in detail in the attempt to assess the feasibility of using rheology as an ink pre-formulation screening marker for optimising the printing quality of porous geometries.

## MATERIALS AND METHODS

### Materials

Paracetamol (PCM) was purchased from Molekula (Molekula, Darlington, UK). Hypromellose, HPMC (METOLOSE® 90SH-4000); MW 270,000 g/mol; 4130 mPas; methoxy content 22–24%; hydroxypropoxy content 8.5–10.5%) was purchased from Shin-Etsu (Shin-Etsu Chemical Co., Ltd., Tokyo, Japan).

### Preparation of Semi-Solid Inks

Semi-solid inks were prepared by mixing different PCM to HPMC ratios in deionised water with the formulas shown in Table I. The aqueous solubility of PCM in water was measured by adding an excess amount of PCM in 20 mL of deionised water (Supplementary Materials Figure S1). The solution was stirred at 300 rpm for 24 h at room temperature and filtered through 0.45 µm nylon membrane filter before analysis at 243 nm. A range of drug loadings covering from below to above the aqueous solubility of PCM in water (Table I), was selected to be incorporated in the inks. For the ink preparation, PCM was first sieved through a stainless-steel test sieve of pore sizes 63, 100 and 250 µm. The sizes of PCM were controlled over the range of < 63, 90–100 and 180–250 µm by further sieving with stainless steel test sieves of pore sizes 90 and 180 µm. However, it should be noted that although the drug particles were controlled using the sieves with defined mesh sizes, there was still a small population of particles with sizes outside of the range, due to the nature of sieving. The inks were prepared by adding PCM in 20 mL of deionised water and stirred for 2 h at room temperature. Subsequently, HPMC was added slowly to be dispersed and dissolved to form a homogenous semi-solid mass under mechanical stirring. As the addition of HPMC may affect the aqueous solubility of PCM, a polarised light microscope was used to examine the inks in order to identify the solubility limit of PCM after the addition of HPMC. A loading of 1.6% w/v PCM was identified as the maximal PCM loading in the current study to prevent any precipitation of PCM particles caused by supersaturation (Supplementary Materials Figure S2). All printing inks were stored in a tightly closed container at room temperature for a minimum period of 15 h before analysis and printing. No significant particle size changes were observed during this period for all inks (data not shown).

**Table I:** Ink Formulations Used in the Study and the Particle Size of PCM Used to Prepare the Inks (PCM 5 and PCM 10) Containing Solid Drug Particles

Formulation code	PCM loading (% w/v)	Particle size of PCM incorporated (µm)	HPMC (% w/v)
HPMC placebo	0.0	-	15
PCM 0.8	0.8	*Fully dissolved	
PCM 1.6	1.6	*Fully dissolved	
PCM 5S	5.0	< 63	
PCM 5M	5.0	90–100	
PCM 5L	5.0	180–250	
PCM 10S	10.0	< 63	
PCM 10M	10.0	90–100	
PCM 10L	10.0	180–250	

### Characterisation of Semi-Solid Inks

#### Rheological Characterisation of Inks

Rheological properties of prepared semi-solid ink were analysed using a Discovery HR-2 hybrid rheometer (TA Instruments, Delaware, USA) fitted with a Peltier plate and stainless-steel cone plate (diameter of 40 mm). Shear viscosity was evaluated by applying a stepwise shear rate ramp from 0.01 to 100 Hz. Storage modulus (G’) and loss modulus (G”) were determined by oscillatory linear strain sweep test from 0.01 to 500% at a constant frequency of 1 Hz and oscillatory linear frequency sweep test from 0.01 to 100 Hz at a constant strain of 1%. All data were analysed using Trios v5.1.1.46572 software (TA Instruments, Delaware, USA). All tests were conducted in triplicate.

### Preparation of 3D Printed Geometries

The semi-solid ink was transferred to a 2.5 mL extrusion syringe with a tapered extrusion tip (22G, ID 0.413 mm). The filled syringe was then transferred to a piston extrusion-based 3D printer (BioX, Cellink Life Sciences, Gothenburg, Sweden) to fabricate the designed 3D construct on a glass build plate at room temperature. The G-code of the designed structure was generated from the predesigned computer-aided design (CAD) model with the dimensions of 20 × 20 × 3 mm with 7 layers of 25% infill. The extruded threads of inks during printing are referred to as ‘filaments’. The selection of this particular infill was to ensure that the spreading effects of the inks on the overall printed structure could be studied. At any other higher infills, the print paths merged either during printing or the drying.

### Printing Quality Assessments of the 3D Printed Geometries

#### Printed Structural Analysis: SSE Extruded Single-Layer Filament Lateral Width and Printed Seven-Layer Geometry Pore Area Measurement

**Fig. 1:**
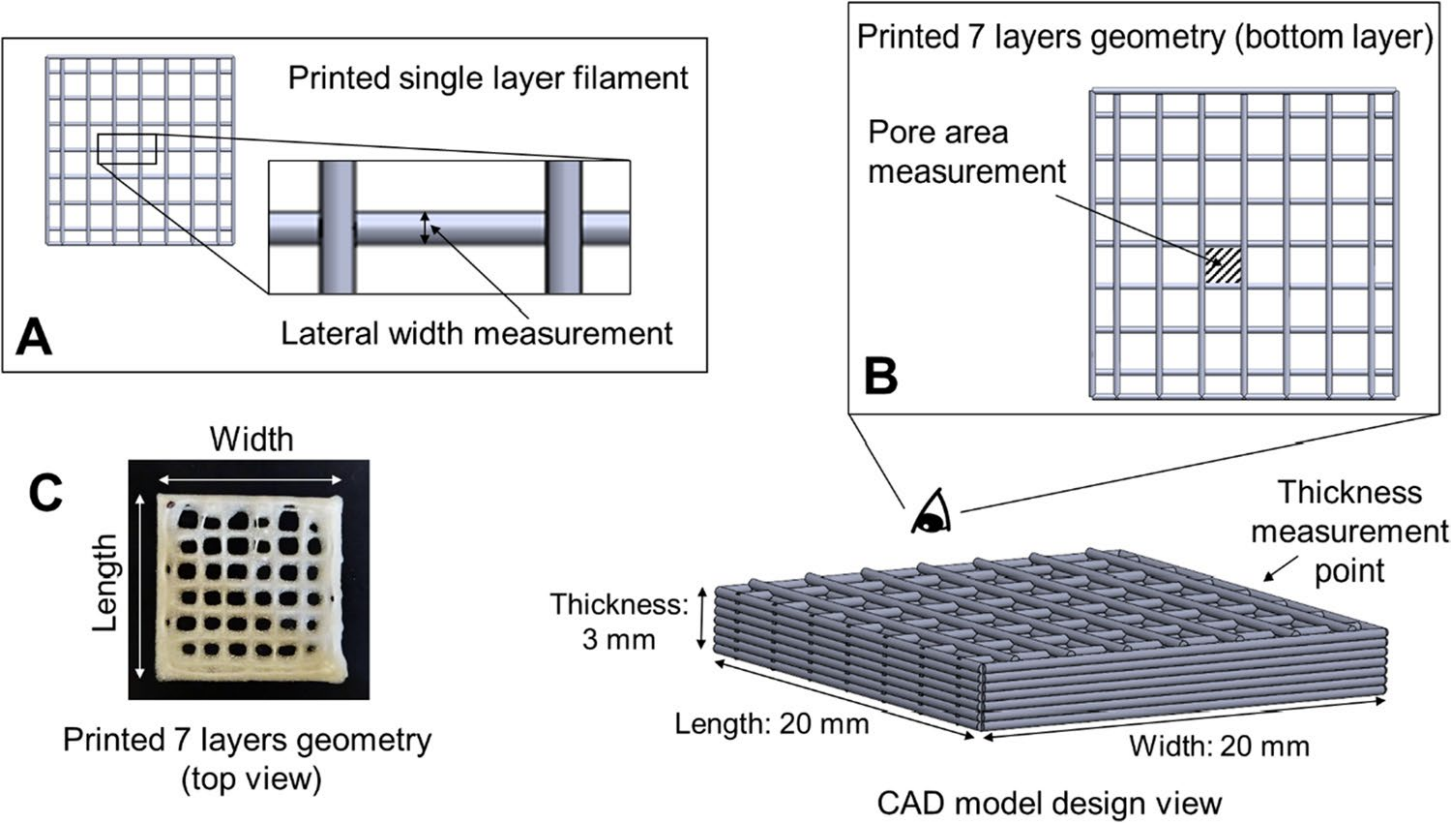
Illustration of (**A**) lateral width measurement of printed single-layer filament, (**B**) pore area and thickness measurements of printed seven-layers geometries and (**C**) printed geometry post 24 h drying.

The single-layer of CAD model was printed to measure the lateral width of the single-layer filament (Fig. 1A). The extrusion rate applied was adjusted individually based on the properties of semi-solid ink to obtain a single filament with a lateral width of 0.413 ± 0.010 mm upon microscopic observation at the fifth-minute post-printing. Seven-layer geometries were then printed using the optimised extrusion rate (as in the single-layer filament printing) and subjected to the pore area measurement (Fig. 1B) throughout the drying process. The reported pore areas of the printed geometries were measured from the pores at the bottom layers of the geometries (in contact with the glass build plate). The drying process of single-layer filaments and seven-layers geometries was captured using a microscope Linkam Imaging Station (Linkam Scientific Instruments, Tadworth, UK). The lateral width of single-layer filament and pore area of seven-layers geometries were further analysed with ImageJ 1.52a software (Wayne Rasband, National Institutes of Health, USA). The change in lateral width and pore area was calculated according to Eqs. 1 and 2. All tests were conducted in triplicate.

1 $$Change\;\;in\;\;lateral\;width\left(\%\right)=\frac{Lateral\;\;width\;\;at\;\;n^{th}minute\;\;after\;\;printing-lateral\;\;width\;\;at\;\;5^{th}minute\;\;after\;\;printing}{Lateral\;\;width\;\;at\;\;5^{th}minute\;\;after\;\;printing}\times100\%$$

2 $$Change\;\;in\;\;pore\;\;area\left(\%\right)=\frac{Pore\;\;area\;\;at\;\;n^{th}minute\;\;after\;\;printing-pore\;area\;\;at\;\;5^{th}minute\;\;after\;\;printing}{Pore\;\;area\;\;at\;\;5^{th}minute\;\;after\;\;printing}\times100\%$$

#### Dimension Measurements of the 3D Printed Geometries

The width and length of printed geometry were measured as per the illustration in Fig. 1C. The overall thickness of printed geometry (as illustrated in Fig. 1B) was measured at a fixed point using a digital vernier calliper after 24 h of drying at room temperature. The moisture content of printed geometries after 24 h of drying was within 1–2.8% as determined using thermogravimetric analysis (data not shown). For each ink formulation, six geometries were printed, and their width, length and thickness were measured (Supplementary Materials Table S1) to confirm the reproducibility of the printing. The dimensional fidelity of the printed geometry, defined in this study as the width, length and thickness of the geometry in comparison to the targeted width, length and thickness in the CAD model, was expressed in percentage as shown in Eq. 3.

3 $$Dimension\;\;fidelity\left(\%\right)=\frac{Dimension\;\;of\;\;dried\;\;printed\;\;geometry}{Dimension\;\;of\;\;CAD\;\;model}\times100\%$$

### Physicochemical Characterisations of the 3D Printed Geometries

#### Scanning Electron Microscopy (SEM)

The surface (top view) of printed geometries was taken with a field emission scanning electron microscope GeminiSEM 300 (Zeiss, Germany). The sample was sputter-coated with gold at 2.2 kV, 5 × 10^–2^ mbar and 55 mm for 30 s (Quorum Technologies, Lewes, UK) prior to image capture.

#### Differential Scanning Calorimetry (DSC)

A differential scanning calorimeter DSC 2500 (TA Instruments, Delaware, USA) was used to evaluate the thermal profiles of printed geometries. Raw materials and printed geometries (approximately 2–5 mg) were crimped in an aluminium pan. The crimped pans were then heated under nitrogen flow (50 mL/min) at a constant scanning rate of 10°C/min from 40°C to 250°C. All tests were conducted in triplicate. Results obtained were analysed by Trios v5.1.1.46572 software (TA Instruments, Delaware, USA).

#### Attenuated Total Reflection-Fourier Transform Infrared (ATR-FTIR)

ATR-FTIR spectra of raw materials and printed geometries were recorded using Bruker Vertex 70 Fourier-Transform Infrared Spectrometer (Serial no. GI003417) equipped with diamond ATR accessory (Specac’s Golden Gate™) over a wavenumber range of 500 cm^−1^ to 4000 cm^−1^ with a resolution of 2 cm^−1^ and 128 scans. Spectra were analysed and illustrated using OPUS software version 7.8.

### Statistical Analysis

All data were expressed as the mean ± SD of three or six determinations. Independent t-test and non-parametric Spearman’s rho correlations analysis were conducted to determine the statistical significance at a 0.05 significance level (p < 0.05) and 0.01 significance level (p < 0.01) respectively. Statistical analysis was conducted using IBM® SPSS® Statistics version 23.

## RESULTS AND DISCUSSION

### Ink Characterisation

**Fig. 2:**
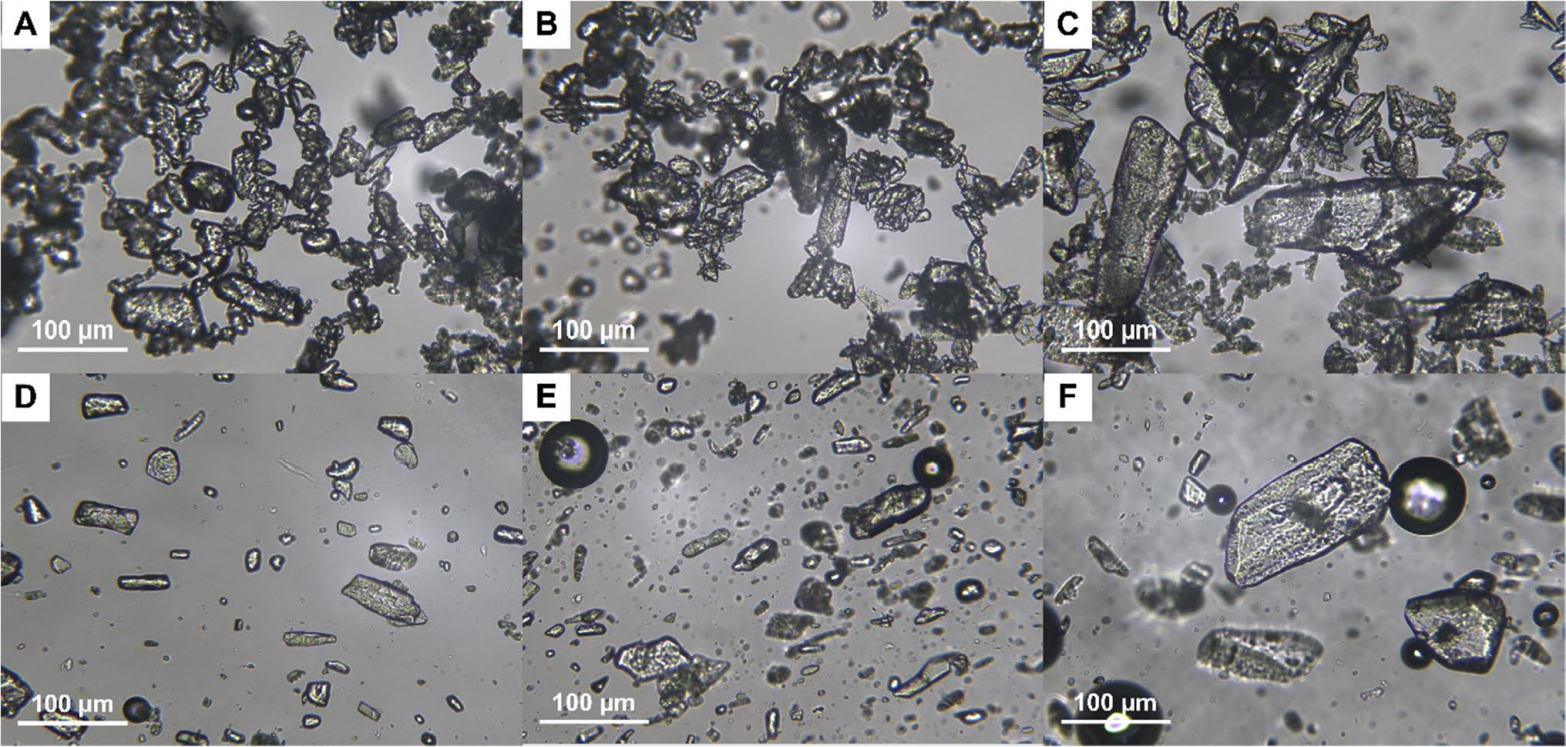
Light microscopic images of PCM particles suspended (5% w/v) in deionised water with particle sizes (**A**) < 63 µm, (**B**) 90–100 µm and (**C**) 180–250 µm; and PCM particles suspended (5% w/v) in inks with particle size (**D**) < 63 µm (PCM 5S), (**E**) 90–100 µm (PCM 5M) and (**F**) 180–250 µm (PCM 5L).

From the solubility study, it was established that at 1.6% w/v PCM concentration in the ink formulation, all drug particles were fully dissolved. Above this drug concentration, i.e., at 5 and 10% w/v, solid drug particles would be present in the ink due to the dissolved drug concentration in the ink reaching its saturation concentration. The morphology of the drug particles in the inks was studied using a light microscope. As shown in Fig. 2A-C, aggregation of undissolved PCM particles with irregular shapes occurred in the aqueous suspensions of PCM 5S, M, L, regardless of the particle size range. It is possible that some of the PCM particles were recrystallised from solution-mediated phase transformation in which dissolved PCM reached its supersaturation, followed by nucleation and growth (21). Subsequent addition of HPMC into these samples containing PCM above its aqueous solubility (i.e. containing undissolved PCM particles) led to the less aggregation/agglomeration of PCM particles in comparison to samples without HPMC, as shown in Fig. 2D-F. Undissolved PCM particles were observed to disperse freely in the HPMC ink. This could be attributed to the adsorption of HPMC on the surface of PCM particles, hence reducing the particle aggregation through the steric repulsion (22).

**Fig. 3:**
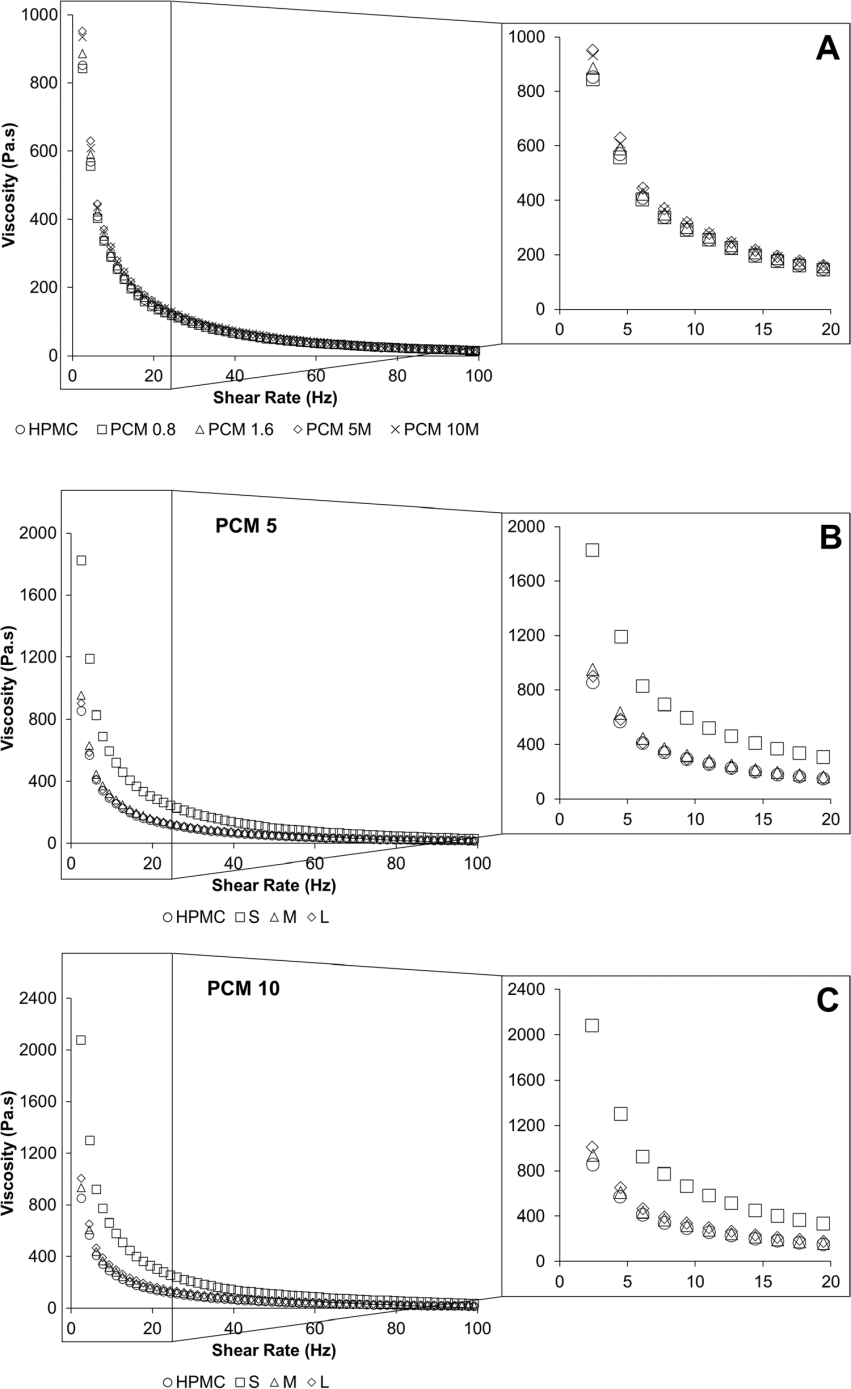
The comparisons of the shear viscosities of the drug-containing inks (**A**) the effect of drug loading; the effect of drug particle size at a PCM loading of (**B**) 5% w/v and (**C**) 10% w/v.

For all inks (without and with solid drug particles), the shear viscosity of the inks decreases with increasing the shear rate, indicating the shear-thinning behaviour, thus demonstrating non-Newtonian behaviour as shown in Fig. 3. At low shear rates, the particle size showed a more profound influence than PCM loading on the ink viscosity. It was noticed that at the lowest shear rate, the viscosities of PCM 5M and PCM 10M were slightly higher than PCM 0.8 and PCM 1.6 (Fig. 3A) which could be due to the presence of solid PCM particles in the inks (23). However, for particle-containing inks (PCM 5 and PCM 10), multiple factors including drug loading, particle size, size distribution and particle shape could lead to a complex effect on the rheological properties of ink containing solid drug particles. As seen in Fig. 3B-C, at low shear rates, the highest viscosity was identified when the PCM particles with the smallest size were incorporated. At the same PCM concentration, the smaller the particle size, the higher number of PCM particles would be present in the ink. It is likely that the higher number of particles in the ink produced an increased resistance to flow and increased particle–particle interaction which led to high viscosity (24).

To further understand the viscoelastic behaviour of the inks, oscillatory rheological tests were performed. Oscillatory amplitude sweep experiments were first carried out to identify the linear viscoelastic (LVE) region which was between 0–10% strain at a frequency of 1 Hz. At this frequency, for all inks the storage modulus values (G’) are greater than the loss modulus (G”) value (data not shown). Both G’ and G” of the inks increase with increasing drug loading. As the strain increased, the structure within the ink was disrupted at the point of flow oscillation strain where G” became greater than G’. As shown in Table II, flow oscillation strain values decrease with increasing the PCM loading. This change is particularly significant for the inks containing solid drug particles, i.e., PCM 5 and PCM 10. Based on Spearman’s rho correlation analysis (with a value of -0.91), the flow oscillation strains of the inks are significantly related to their drug loading (p < 0.01). This could be attributed to the presence of PCM which weaken the intramolecular force in the HPMC gel network.

**Table II:** Rheological Flow Oscillation Strain Values of Inks (Obtained from Oscillatory Amplitude Sweep Tests) and Their G’ and G” Values at an Oscillatory Frequency of 1 Hz (Obtained from Oscillatory Frequency Sweep Tests)

Formulation code	Flow oscillation strain (%) ± SD	Storage modulus (G’, Pa) ± SD	Loss modulus (G”, Pa) ± SD
HPMC*	141.60 ± 1.69	5.08 × 10^3^ ± 1.04 × 10^2^	3.18 × 10^3^ ± 6.33 × 10
PCM 0.8*	138.36 ± 1.09	5.10 × 10^3^ ± 5.68 × 10	3.25 × 10^3^ ± 3.83 × 10
PCM 1.6*	138.76 ± 3.35	5.42 × 10^3^ ± 2.44 × 10^2^	3.44 × 10^3^ ± 1.18 × 10^2^
PCM 5S	133.48 ± 0.39	5.88 × 10^3^ ± 1.91 × 10^2^	3.70 × 10^3^ ± 7.83 × 10
PCM 5M	129.13 ± 0.83	5.12 × 10^3^ ± 9.82 × 10	3.35 × 10^3^ ± 6.45 × 10
PCM 5L	130.08 ± 0.15	5.50 × 10^3^ ± 5.52 × 10	3.59 × 10^3^ ± 3.92 × 10
PCM 10S	122.61 ± 2.26	6.07 × 10^3^ ± 6.43 × 10	3.89 × 10^3^ ± 3.47 × 10
PCM 10M	119.40 ± 0.97	6.26 × 10^3^ ± 4.73 × 10	4.01 × 10^3^ ± 1.16 × 10
PCM 10L	118.77 ± 1.28	5.83 × 10^3^ ± 4.50 × 10^2^	3.79 × 10^3^ ± 2.37 × 10^2^

^*^ Inks with no solid drug particles

**Fig. 4:**
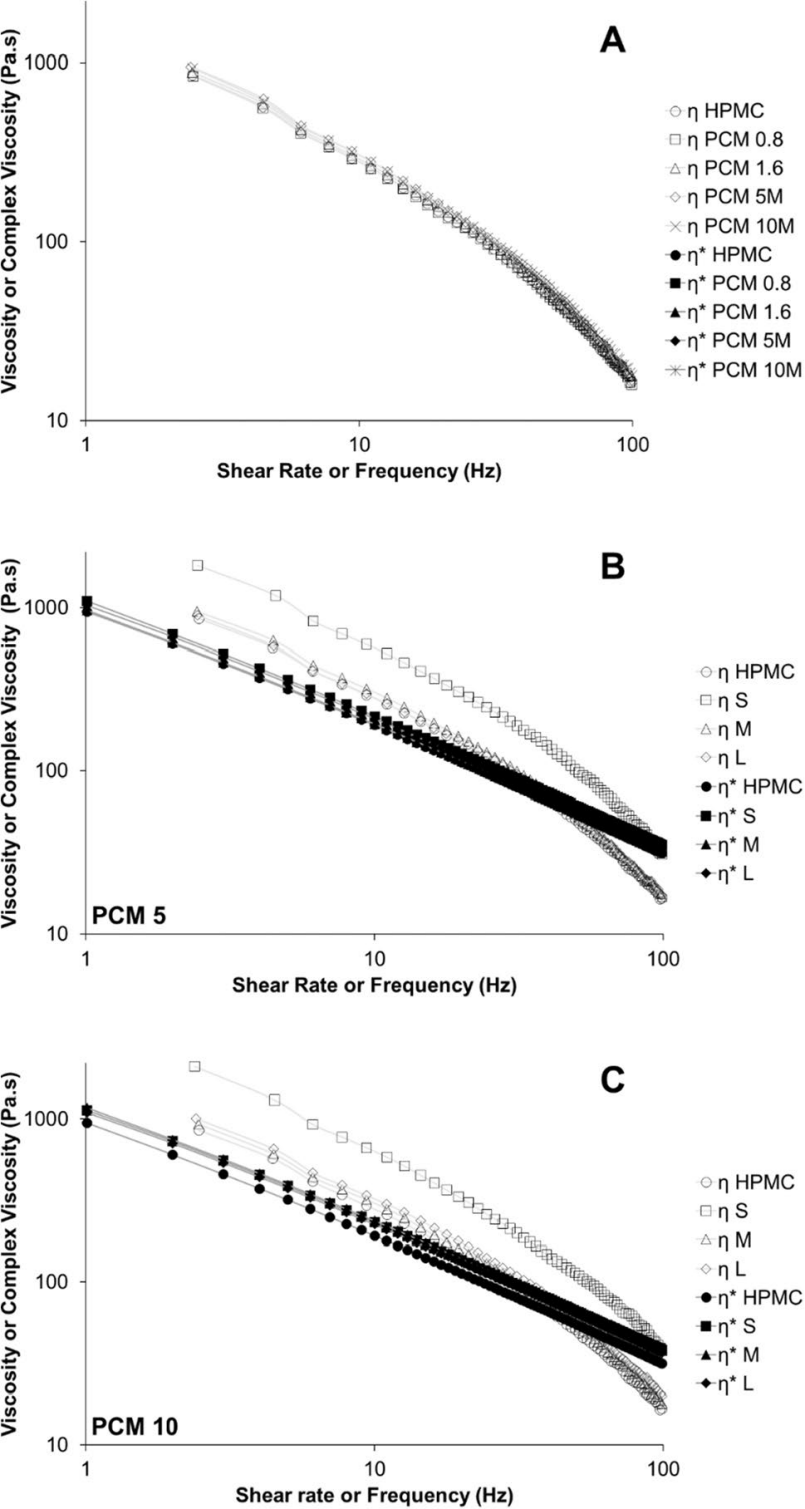
Shear viscosity (η) (obtained using rotational shear viscosity measurements) and complex viscosity (η*) (obtained using oscillatory frequency sweep tests) plots for (**A**) inks with different PCM loading (all PCM particles are in the mid-size range, 90–100 µm); (**B**) inks with 5% PCM loading and different particle sizes; (**C**) with 10% PCM loading and different particle sizes.

The oscillatory frequency sweep tests were used to measure the complex viscosity (η*). At the strain of 1% (within the LVE region), all inks have a greater G’ value than the G” (data shown in Table II and Supplementary Materials Figure S3). As shown in Fig. 4, η* of all the inks decreases with increasing oscillatory frequency, confirming their shear-thinning behaviour (25). This result agrees well with the shear viscosity (η) analysis (Fig. 3). In the literature, shear viscosity was more commonly employed to predict the printability of inks in SSE printing (4, 26, 27), but the correlation between complex viscosity and printability of SSE inks has not been explored.

As described by the Cox-Merz rule that the frequency dependence of complex viscosity should be proportional to the shear rate dependence of shear viscosity in non-Newtonian fluids (28). Therefore, according to the Cox-Merz rule, either shear or complex viscosity measurements should provide similar outcomes in terms of the correlations between ink viscosity and printability. However, the data shown in Fig. 4 deviates from the Cox-Merz empirical relationship where the trends of shear viscosity and complex viscosity diverged when the shear rate or frequency is above 10 Hz. This result indicates that shear viscosity and complex viscosity cannot be used as interchangeable indicators for predicting the printability of SSE inks.

### Printing Optimisation

The optimisation of the SSE printing was performed by defining the optimal extrusion rate at which the ink can be consistently extruded to produce a filament with consistent lateral width. As the inks showed different rheological properties, to produce filaments with a consistent lateral width that is close to the nozzle diameter, individualised optimisation was required for the inks at specific drug loading and containing PCM particles with different particle sizes (data shown in Table III). In general, the required extrusion rate decreased when PCM particles were present in the formulated inks (PCM 5S, M, L and PCM 10S, M, L). This trend agrees well with the trend of the measured flow oscillation strain data of the inks. However, the extrusion rates required for the inks showed no clear trend/correlation with particle size.

**Table III:** The Effects of Drug Loading on the Extrusion Rates for SSE Printing, Dimensional Fidelity of the Seven-Layer Printed Geometries After 24 Hours Drying at Room Temperature. (n = 6)

Formulation code	Extrusion rate (µL/s)	Dimensional fidelity after drying (width/length/thickness of dried printed geometry to CAD model) (%)		
		Width	Length	Thickness
HPMC*	5.00	96.85 ± 0.54	97.24 ± 0.61	15.00 ± 1.01
PCM 0.8*	4.30	97.39 ± 1.13	98.04 ± 0.32	15.72 ± 2.53
PCM 1.6*	4.30	97.53 ± 1.00	98.29 ± 0.69	15.00 ± 3.42
PCM 5S	2.05	97.36 ± 1.37	96.86 ± 0.38	13.94 ± 0.65
PCM 5M	4.00	98.63 ± 0.58	98.29 ± 0.36	14.22 ± 0.58
PCM 5L	3.50	98.93 ± 1.04	98.31 ± 0.90	13.89 ± 1.87
PCM 10S	4.00	97.28 ± 0.51	97.23 ± 0.59	17.33 ± 2.52
PCM 10M	3.00	97.54 ± 0.35	97.08 ± 0.74	12.50 ± 1.52
PCM 10L	4.00	97.41 ± 0.54	97.44 ± 0.42	15.06 ± 2.14

^*^ Inks with no solid drug particles present

### Physicochemical Characterisations of SSE 3D Printed Geometries

**Fig. 5:**
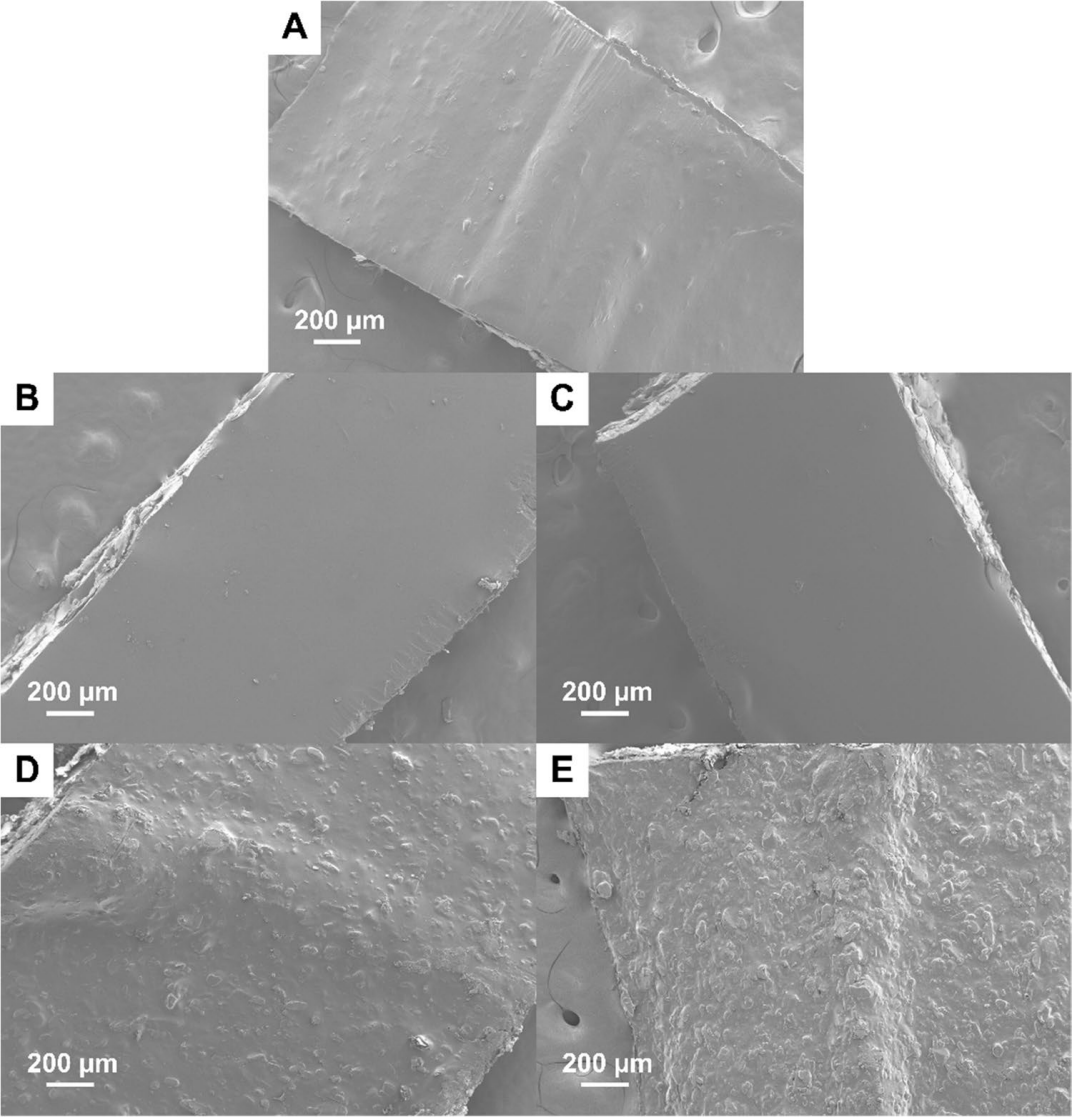
SEM images of the surface morphology (top view) of dried seven-layer printed geometry of formulation (**A**) HPMC, (**B**) PCM 0.8, (**C**) PCM 1.6, (**D**) PCM 5M and (**E**) PCM 10M.

**Fig. 6:**
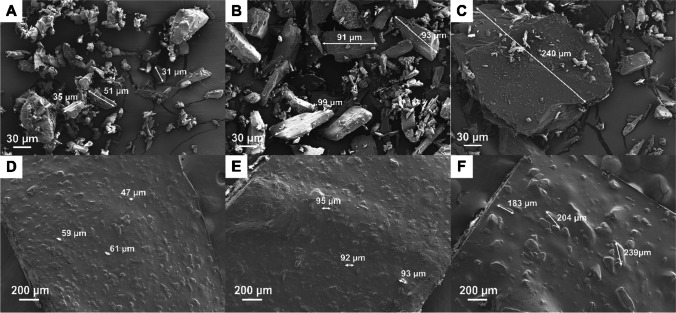
SEM images of the morphology of PCM powder with the particle size of (**A**) S (< 63 µm), (**B**) M (90–100 µm) and (**C**) L (180–250 µm). Note that there were small populations of PCM particles sized out of the mentioned range due to the nature of sieving. Surface morphology (top view) of dried printed PCM 5 geometries with the PCM particle size of (**D**) S (< 63 µm), (**E**) M (90–100 µm) and (**F**) L (180–250 µm).

As shown in Fig. 5A, the seven-layer placebo print showed a low level of roughness of the top surface, which may be due to surface drying causing the formation of self-aggregations of HPMC (29). Interestingly, the prints of PCM 0.8 and PCM 1.6 (below PCM saturation concentration in the inks) show a reduction in surface roughness compared to the surfaces of the placebo prints (Fig. 5B-C). Such observations could be attributed to the reduction in HPMC self-aggregation when PCM molecules were present within the HPMC network (29). However, further increasing the PCM loading to above its saturation concentration in the ink, the surface roughness of the prints significantly increases (Fig. 5D-E). The roughness could be mainly attributed to the presence of solid PCM particles. This can be partially confirmed by the particle size comparison demonstrated in Fig. 6. The sizes of the particles seen on the surfaces of dried PCM 5 and PCM 10 prints are in the same dimensional range as the PCM particles used to prepare the inks.

**Fig. 7:**
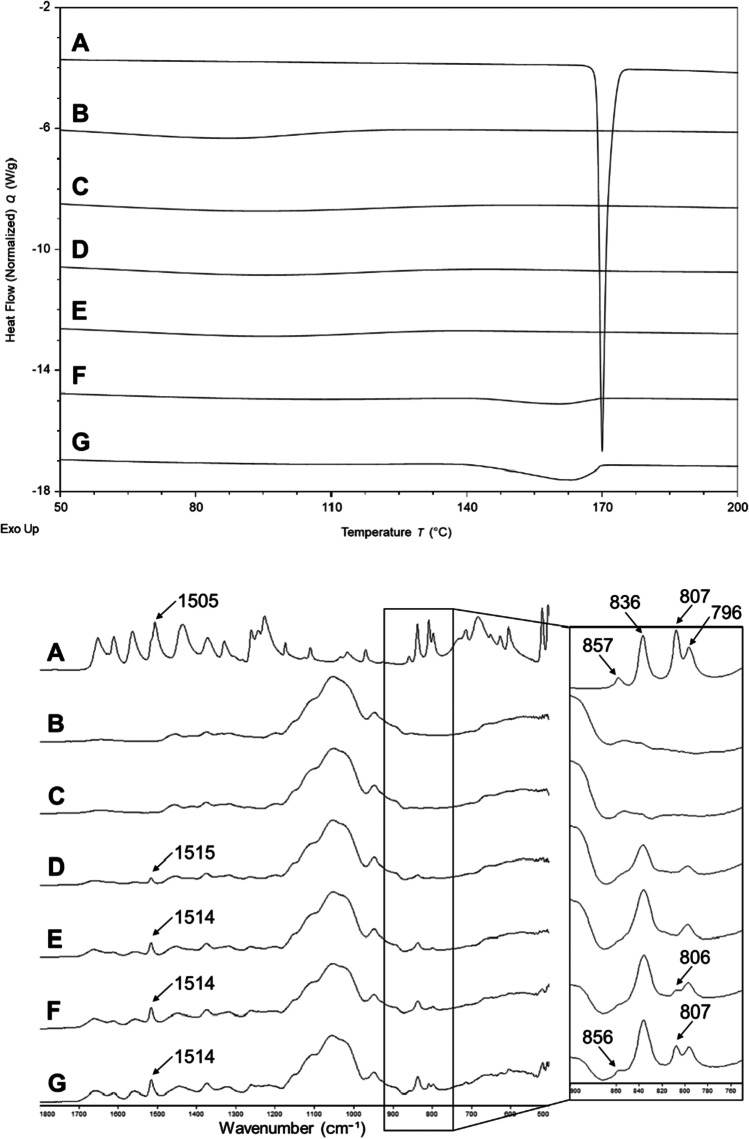
DSC thermograms (top) and ATR-FTIR spectra (bottom) of (**A**) crystalline PCM powder, (**B**) HPMC powder, dried geometries of (**C**) HPMC, (D) PCM 0.8, (**E**) PCM 1.6, (**F**) PCM 5M and (**G**) PCM 10M. Arrows in the ATR-FTIR spectra indicate the characteristic peaks of crystalline PCM form I.

The DSC results of the PCM 0.8 and PCM 1.6 prints showed no melting of crystalline PCM, whereas broad and depressed melting between 145–170°C can be seen for PCM 5M and PCM 10M (Fig. 7(top)). This data confirmed the absence of crystalline PCM in PCM 0.8 and PCM 1.6 prints, and the presence of a significant amount of undissolved crystalline PCM particles in PCM 5 and PCM 10 prints. Characteristic absorption bands of crystalline PCM, including 796 and 836 cm^−1^ C-N and C-O stretching can be identified in the ATR-FTIR spectra of all the PCM-containing printed geometries, as shown in Fig. 7D-G (bottom) (30). The peak intensity of characteristic bands of PCM increased with increasing PCM loadings in the inks. In the spectra of PCM 5 and PCM 10 prints, C-H bending at 806–807 cm^−1^ is evident which is a signature of crystalline PCM monoclinic (or Form I) (31). For all printed geometries, the characteristic aromatic ring band of PCM Form I at 1505 cm^−1^ shifted to a higher wavenumber to 1514–1515 cm^−1^. A similar observation of the characteristic band at 1514 cm^−1^ was also reported in the previous dispersion of PCM in EUDRAGIT® E system (31) and attributed to a change of PCM stable Form I to metastable orthorhombic (or Form II) after recrystallisation in the solid dispersion (32, 33). Therefore, it is possible that a mixture of different polymorphs of PCM was present in prints of PCM 5M and PCM 10M, as both showed the characteristic peaks of monoclinic and orthorhombic polymorphs of crystalline PCM. However, it should be noted that in the ATR-FTIR experiment the signal arises from absorption of the evanescent ray, which penetrates the surface of the sample to a depth of the order of 1 µm. The particles are much larger than this so it may be that the polymorph is only located in some regions of the particle surfaces. Since there is no evidence of recrystallisation leading to the growth of large drug crystals, the observation of some IR signal from the orthorhombic form was probably due to friction between the particles causing small amount of drug recrystallisations on the particle surfaces. Similar polymorphic transformations have been reported during milling processes (34).

### Printing Quality Assessment of SSE 3D Printed Geometries

The percentage change in the width, length and thickness of the seven-layer print against the CAD design was used as an indicator for dimensional fidelity (Table III). All printed geometries achieved an average of 96–98% dimensional fidelity for width and length. However, it was demonstrated that regardless of drug loading and drug particle size in the inks, the dimensional fidelity of thickness was in the order of 12–17%, indicating significant ‘sagging’ of the printed structures. As the focus of this study is not to produce an optimal print but to enrich the understanding of the correlation between ink properties and the printing quality, we did not further optimise the formulation by introducing other additives to improve printability and structural integrity, such as reported in the literature (2, 35).

**Fig. 8:**
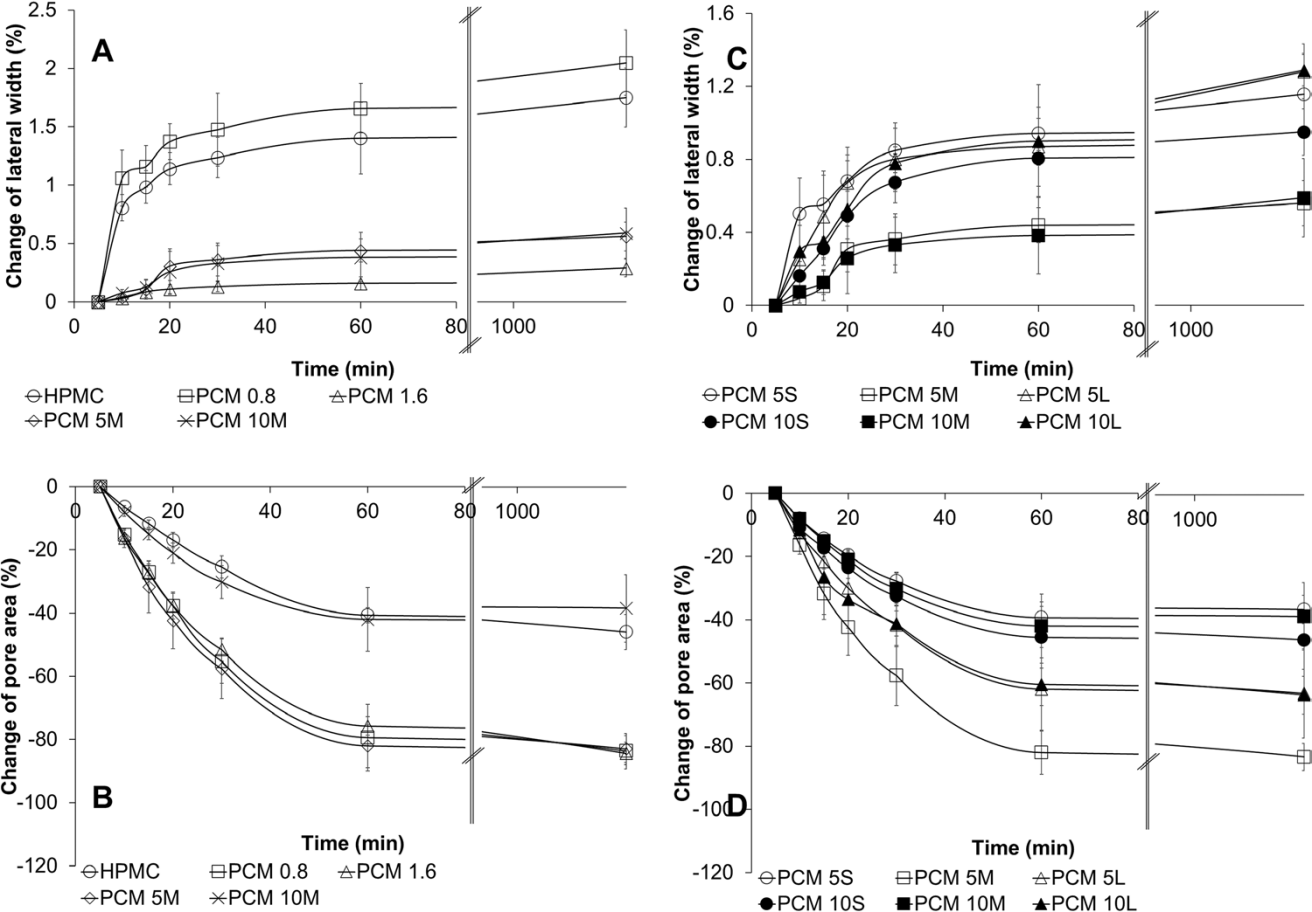
Printing quality evaluation by comparing the (**A**) filament lateral width changes of single-layer prints and (**B**) pore area changes of seven-layer prints based on the effect of drug loading at a constant particle size and (**C**) filament lateral width changes of single-layer prints and (**D**) pore area changes of seven-layer prints based on the effect of drug particle size throughout the drying process.

To investigate further the printing quality, the change of filament lateral width during drying within a single printed layer and the change of pore areas in the middle of the bottom layer of the seven-layer print (as illustrated in Fig. 1) were measured. The resistance to deformation, such as spreading and sagging, during the drying process, was assessed by calculating the percentage of change in the filament lateral width and the pore area. The results were summarised in Fig. 8. For the single-layer placebo prints, the structural deformation was observed as the horizontal spreading of the filament induced by factors such as surface tension, the flow and the mechanical changes of ink during drying (36). Prints of placebo and PCM 0.8 showed the highest increases in lateral width, 2%, among all inks. An increase in PCM loading up to 1.6% w/v which contains no particles present and PCM 5S, M, L and PCM 10S, M, L which contained solid drug particles show a significant reduction (p < 0.05) in lateral width change in comparison to placebo and PCM 0.8 (a detailed comparison of the data can be found in Supplementary Materials Figure S4.). This could be explained by the higher G’ and G” values of PCM 1.6, PCM 5 and PCM 10 inks in comparison to the placebo and PCM 0.8 inks (as shown in Table III), hence the higher ability to resist structural deformation. No significant difference (p > 0.05) was observed between the PCM particle-containing inks (PCM 5 and PCM 10), provided the same PCM particle size was loaded (Fig. 8C), indicating the spreading is more affected by the particle size than drug loading for the inks with solid drug particles. It is worth noting that this observation is limited to the drug loading tested in this study and wider generalisation requires a wider range of high drug loadings being tested. Figure 8C also showed that the single-layer prints of PCM 5M and PCM 10M which contain the narrowest PCM particle size range (based on the dry drug particle size data) show the least change in lateral width of filament compared to those of PCM 5S/L and PCM 10S/L which have the same drug loadings but wider size distribution. Therefore, this result may indicate the uniformity of PCM particle distribution has an effect on minimising the horizontal spreading of single-layer filament prints.

In the case of the printed seven-layer porous geometries, heterogeneity of pore area within the same print was observed during the drying process. It was noted that the pore closure caused by filament merging was locationally dependent. A higher merging tendency was observed at the edges of the prints as a result of the horizontal inhomogeneous drying process (37). This is related to the collapse of the print walls since the reduction in height due to drying requires that the mass of the material in the wall is concentrated at the base. The change in the pore area sampled from the middle of the print, as shown in Fig. 1, is attributed to a thickening of the filaments rather than a contraction in their length, as evidenced by the small changes in print width and length. There is no significant correlation between dimensional fidelity and PCM loading.

Although the change of single-layer filament lateral width in placebo print was high, the changes in pore area after printing and drying print was one of the lowest amongst all tested formulations. This is to be expected if the increase in filament width in the seven-layer samples is due to the collapse of all the layers to the base of the print, as discussed above, since the total mass of solid left on drying will be one-seventh of that from the seven-layer print. The prints with PCM particles (PCM 5S, M, L and PCM 10S, M, L) did not reveal a consistent effect of drug loading on the structural deformation as observed from the reduction in pore area. The seven-layer porous prints of PCM 5S and PCM 10S and 10M showed a similar level of changes in pore area to the placebo prints and were significantly (p < 0.05) less than the rest of the drug-loaded prints (Supplementary Materials Figure S5).

## CONCLUSION

This study investigated the effects of drug loading and particle size (if present) in HPMC-based inks on the printing quality of porous geometries using SSE 3D printing. The results indicated the solid drug particle loading and size can have significant impacts on the shear viscosities of the inks at low shear rates. The highest shear viscosity was observed in the inks containing the highest drug loading and the smallest PCM particles, but the shear viscosity did not show a direct correlation with the optimal extrusion rate (in order to obtain desired initial lateral width of filament) required for each ink formulation. The rheological data showed a significant correlation between drug loading and flow oscillation strain of the inks. A higher drug loading in the inks led to increased oscillatory rheological moduli, particularly the storage modulus, loss modulus and complex viscosity. Inks with higher oscillatory rheological moduli showed higher resistance to pore structural deformation for multi-layer printing. The inclusion of particles in SSE inks has a complex effect on printability and printing quality. The effects depend on particle size, size distribution and particle loading and are not easily predictable. For the inks containing solid particles, oscillatory rheological properties can be potentially used as a pre-screening indicator to predict the printing quality of pore geometry.

## Supplementary Information

Below is the link to the electronic supplementary material.

Supplementary file1 (DOCX 1.97 MB)
