# Supplementary material for: The Effects of Solid Particle Containing Inks on the Printing Quality of Porous Pharmaceutical Structures Fabricated by 3D Semi-Solid Extrusion Printing
Source: Pharm Res. 2022 Jun 3;39(6):1267–79. doi: 10.1007/s11095-022-03299-7 (PMC9197916; doi:10.1007/s11095-022-03299-7)
Supplement: Supplementary file 1 — Supplementary file1 (DOCX 1.97 MB) [file 11095_2022_3299_MOESM1_ESM.docx]

**Supplementary Materials**

**The effects of solid particle containing inks on the printing quality of porous pharmaceutical structures fabricated by 3D semi-solid extrusion printing**

Xin-Yi Teoh^1,2^, Bin Zhang^1^, Peter Belton^3^, Siok-Yee Chan^2^, Sheng Qi^1^*

^1^ School of Pharmacy, University of East Anglia, Norwich, United Kingdom

^2^ School of Pharmaceutical Sciences, Universiti Sains Malaysia, Penang, Malaysia

^3^ School of Chemistry, University of East Anglia, Norwich, United Kingdom

*Corresponding author: [sheng.qi@uea.ac.uk](mailto:sheng.qi@uea.ac.uk)

**List of content:**

**Figure S1.** Calibration curve of PCM in deionised water analysed at 243 nm.

**Figure S2.** Qualitative screening of PCM solubility in the presence of 15% w/v HPMC. Morphology of (A) HPMC, inks with PCM loading of (B) 0.8% w/v (PCM 0.8), (C) 1.6% w/v (PCM 1.6), (D) 2% w/v, (E) 3% w/v and (F) 4% w/v were captured under the polarised light microscope. The presence of birefringence indicates the presence of undissolved PCM particles.

**Figure S3.** Rheological characteristics of prepared inks comparing the storage modulus (G’) and loss modulus (G”) based on (A) the effect of drug loading, (B) the effect of drug particle size at a loading of PCM 5 and (C) the effect of drug particle size at a loading of PCM 10.

**Figure S4.** Printing quality evaluation by comparing the filament lateral width changes of single-layer prints based on (A) the effect of drug loading at a constant particle size and the effect of drug particle size at a drug loading of (B) 5% w/v (PCM 5) and (C) 10% w/v (PCM 10).

**Figure S5.** Printing quality evaluation by comparing the pore area changes of seven-layer prints based on (A) the effect of drug loading at a constant particle size and the effect of drug particle size at a drug loading of (B) 5% w/v (PCM 5) and (C) 10% w/v (PCM 10).

**Table S1.** The effects of drug loading on the thickness, width and length of printed seven-layer geometries after 24 hours of drying at room temperature. (n=6)

**Identification of saturated aqueous solubility of PCM**

The saturated solubility of PCM in water was measured by adding an excess amount of PCM in 20 mL of deionised water. The solution was stirred at 300 rpm for 24 hours at room temperature and filtered through 0.45 µm nylon membrane filter before analysis at 243 nm. The test was conducted in triplicate. The calibration curve of PCM in deionised water is shown in **Figure S1**. The saturated aqueous solubility of PCM was determined at 16.27 ± 1.07 mg/mL.

Figure S1. Calibration curve of PCM in deionised water analysed at 243 nm.

**Qualitative analysis of saturation of PCM in HPMC inks**

As the addition of HPMC may affect the aqueous solubility of PCM in the inks, the inks were subjected to observation under the polarised light microscope to identify the presence of solid PCM particles (if any). **Figure S2** shows the presence of birefringence in the HPMC inks with PCM loading of 3% w/v and above, indicating the presence of undissolved PCM particles within the inks. The saturation of PCM in HPMC ink could be between PCM loading of 2-3% w/v. Subsequent analysis of the printing ink PCM 5M and PCM 10M confirmed the presence of solid PCM particles with the observation of birefringence within the inks.


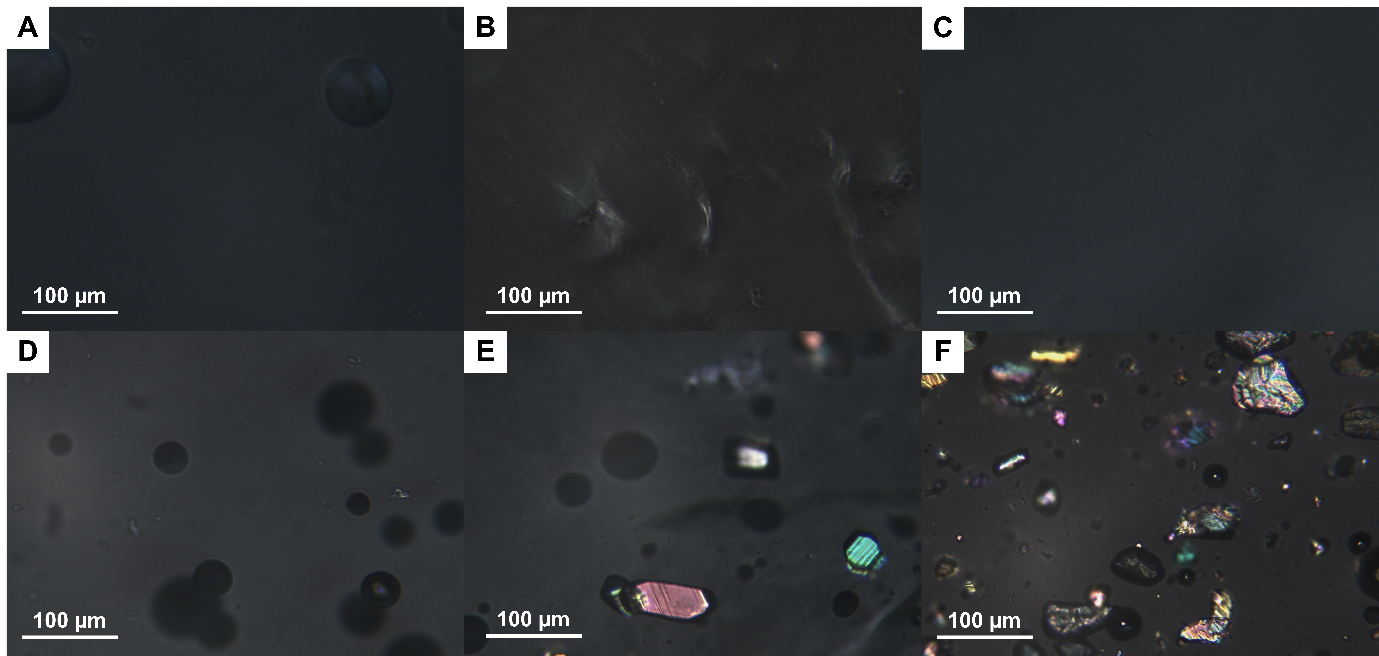


**Figure S2.** Qualitative screening of PCM solubility in the presence of 15% w/v HPMC. Morphology of (A) HPMC, inks with PCM loading of (B) 0.8% w/v (PCM 0.8), (C) 1.6% w/v (PCM 1.6), (D) 2% w/v, (E) 3% w/v and (F) 4% w/v were captured under the polarised light microscope. The presence of birefringence indicates the presence of undissolved PCM particles.

**Oscillatory linear frequency sweep test of inks**

The storage moduli (G’) and loss moduli (G”) of the inks measured by oscillatory linear frequency sweep from 0.01 to 20 Hz at a constant strain of 1% are shown in **Figure S3**. All tests were conducted in triplicate. Increasing PCM loading led to increased G’ and G” values, which is particularly evident for PCM 10S, M, L inks.


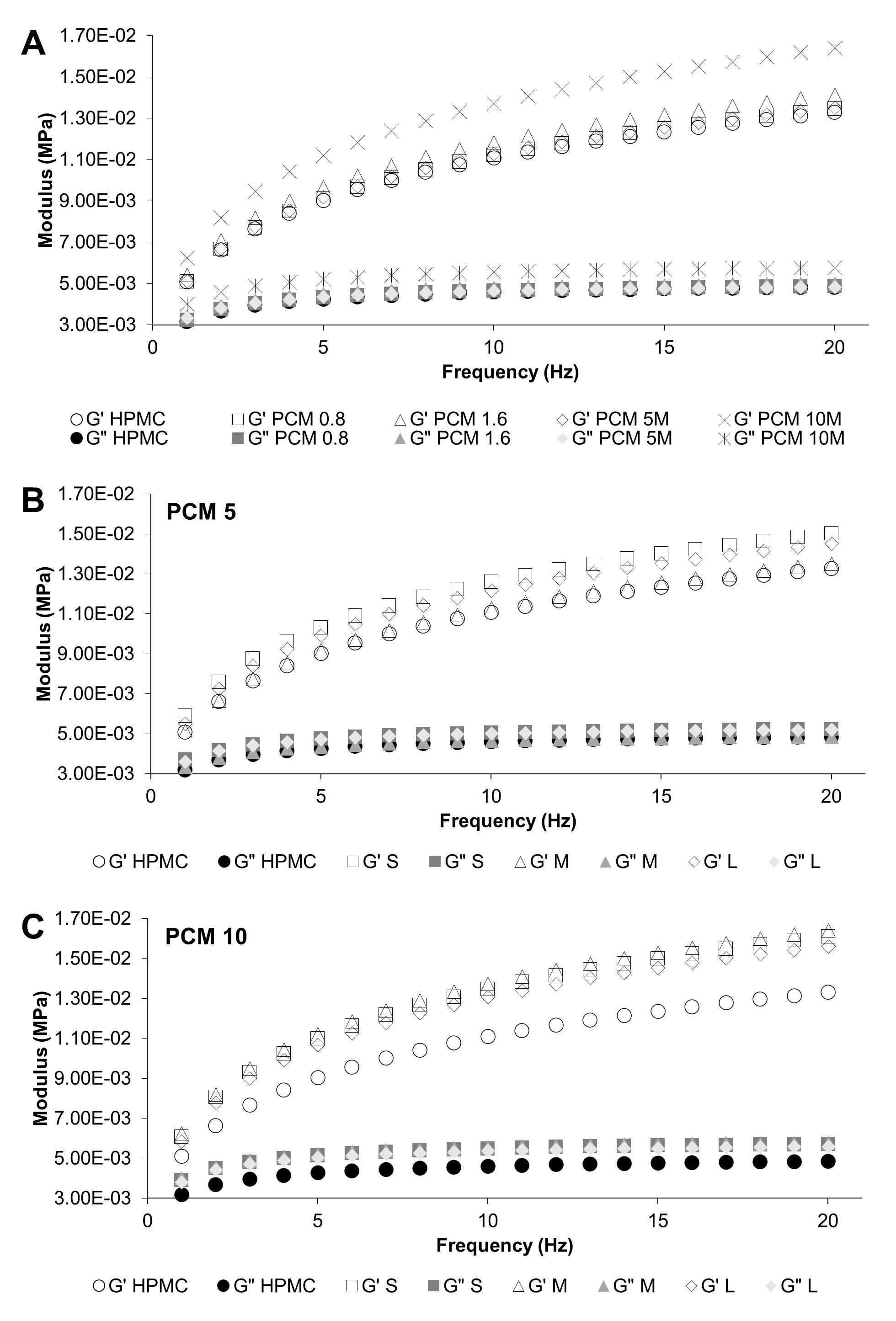


Figure S3. Rheological characteristics of prepared inks comparing the storage modulus (G’) and loss modulus (G”) based on (A) the effect of drug loading, (B) the effect of drug particle size at a loading of PCM 5 and (C) the effect of drug particle size at a loading of PCM 10.

**Printing quality assessment of SSE 3D printed filaments and geometries**

Analysis of dimensions and printing qualities of the SSE 3D printed filaments and porous geometries are summarised in Table S1, Figure S4 and Figure S5. As seen in Table S1, the measured width and length of the outer dimensions of the SSE 3D printed seven-layer geometries after drying are within a range of 19.37-19.79 mm and 19.37-19.66 mm, respectively, which highly resemble the CAD model design (20 mm). However, significant reductions in thickness of the printed geometry (within the range of 0.38-0.52 mm) were observed in all ink formulations. The drug loading and drug particle size showed little effect on the degree of reduction in thickness.

**Table S1.** The effects of drug loading on the thickness, width and length (outer dimension) of printed **seven-layer** geometries after 24 hours of drying at room temperature. (n=6)

| **Formulation code** | **Width (mm)** | **Length (mm)** | **Thickness (mm)** |
| --- | --- | --- | --- |
| HPMC* | 19.37 ± 0.11 | 19.45 ± 0.12 | 0.45 ± 0.03 |
| PCM 0.8* | 19.48 ± 0.23 | 19.61 ± 0.06 | 0.47 ± 0.08 |
| PCM 1.6* | 19.51 ± 0.20 | 19.66 ± 0.14 | 0.45 ± 0.10 |
| PCM 5S | 19.47 ± 0.27 | 19.37 ± 0.08 | 0.42 ± 0.02 |
| PCM 5M | 19.73 ± 0.12 | 19.66 ± 0.07 | 0.43 ± 0.02 |
| PCM 5L | 19.79 ± 0.21 | 19.66 ± 0.18 | 0.42 ± 0.06 |
| PCM 10S | 19.46 ± 0.10 | 19.45 ± 0.12 | 0.52 ± 0.08 |
| PCM 10M | 19.51 ± 0.07 | 19.42 ± 0.15 | 0.38 ± 0.05 |
| PCM 10L | 19.48 ± 0.11 | 19.49 ± 0.08 | 0.45 ± 0.06 |

* No solid drug particles present

The printing quality of the single printed layer was evaluated by measuring the change in filament lateral width. As seen in Figure S4, the print of PCM 0.8 showed the highest increase in lateral width among all the prints followed by the placebo print. No significant difference was identified between the prints of placebo and PCM 0.8. Further increase in the PCM loading up to 1.6% (PCM was fully dissolved), 5% and 10% (PCM particles were present) w/v showed a significant reduction (p<0.05) in lateral width change compared to the placebo and PCM 0.8.

In the study of seven-layer porous geometries, pore area changes were measured to deduce the printing quality of inks (Figure 5S). An increase in the PCM loading of the ink showed a consistent significant reduction in pore area in comparison to the placebo provided PCM loaded was fully dissolved in the ink. At a higher PCM loading where drug particles were present in the inks (PCM 5S, M, L and PCM 10S, M, L), no consistent significant difference was reported with the variation of drug loading and particle size on the pore area reduction compared to the placebo print


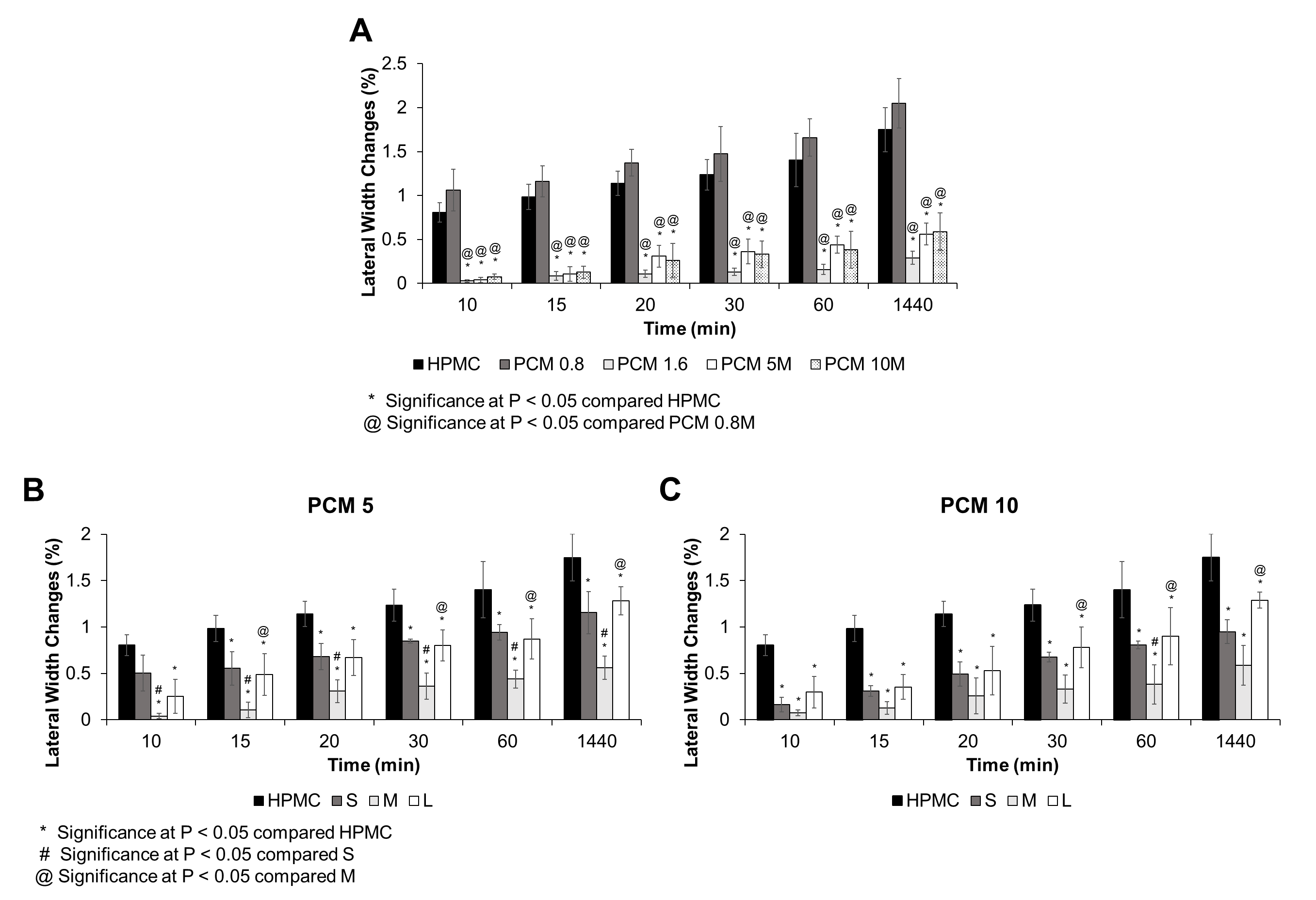


**Figure S4.** Printing quality evaluation by comparing the filament lateral width changes of single-layer prints based on (A) the effect of drug loading at a constant particle size and the effect of drug particle size at a drug loading of (B) 5% w/v (PCM 5) and (C) 10% w/v (PCM 10).


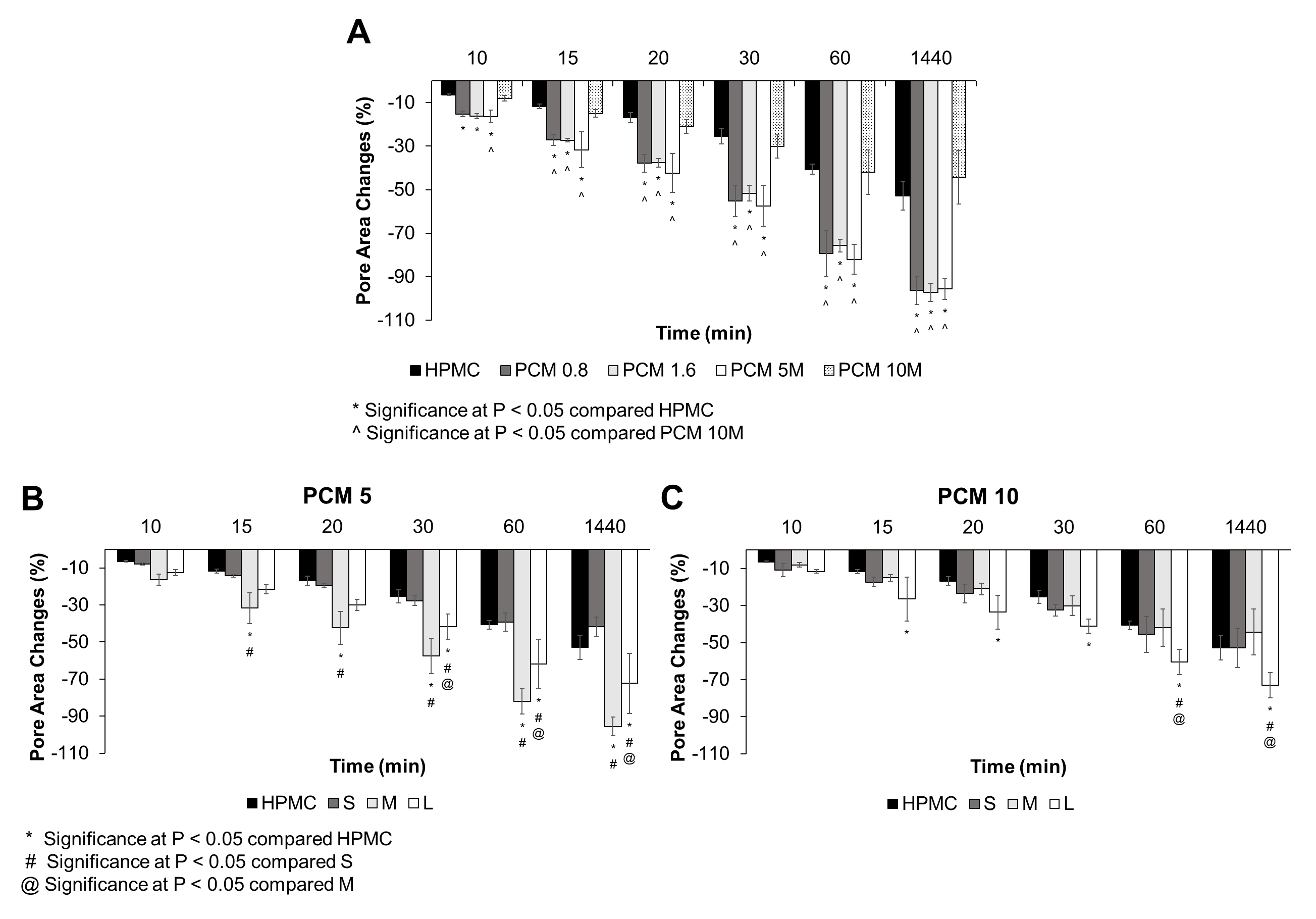


**Figure S5.** Printing quality evaluation by comparing the pore area changes of seven-layer prints based on (A) the effect of drug loading at a constant particle size and the effect of drug particle size at a drug loading of (B) 5% w/v (PCM 5) and (C) 10% w/v (PCM 10)
